# VFMSS findings in elderly dysphagic patients: our experience

**DOI:** 10.1186/1471-2482-13-S2-S54

**Published:** 2013-10-08

**Authors:** Alfonso Reginelli, Francesca Iacobellis, Lucia Del Vecchio, Luigi Monaco, Daniela Berritto, Graziella Di Grezia, Eugenio Annibale Genovese, Melchiore Giganti, Salvatore Cappabianca

**Affiliations:** 1 Department of Internal and Experimental Medicine, Magrassi-Lanzara, Institute of Radiology, Second University of Naples, Naples, Italy; 2 Department of Internal and Experimental Medicine, Magrassi-Lanzara, Institute of Gastrointestinal Surgery, Second University of Naples, Naples, Italy; 3 University of Cagliari, Department of Radiology, Cagliari, Italy; 4 Department of Surgical Sciences, University of Ferrara, Ferrara, Italy

## Abstract

**Background:**

Dysphagia consists in alteration of the swallowing mechanism, due to different pathological conditions, which can occur at different levels. The exact prevalence of dysphagia is unclear, even if some AA suggest that 15% of the elderly population is affected. Aim of this study is to analyze the main VFMSS findings in elderly dysphagic patients.

**Materials and methods:**

The VFMSS of 59 elderly dysphagic patients (32 women, 27 men, ranging in age from 68 to 89 years, mean 81 years) who undergone speech therapy assessment and videofluoromanometric (VFM) investigation of the swallowing process at our institution from January 2011 and December 2012, were retrospectively reviewed.

**Results:**

In the oral phase the preparation and the initial stage of swallowing should be explored by videofluoroscopy evaluating the ability to contain food in mouth and to form a bolus and whether there is an inadequate convergence of Passavant's ridge with preswallowing aspiration. In the pharyngeal phase is necessary to evaluate at videofluoroscopy if there is penetration and/or aspiration and the efficacy of laryngeal closure should be assessed too.

The major manometric indicators are: proximal pharyngeal pressure (mmHg), distal pharyngeal pressure (mmHg), relaxation and coordination of upper esophageal sphincter (UES). In the esophageal phase is important to evaluate the esophageal motility and the presence of peristalsis.

The manometric parameters used for LES were resting pressure, total length and percentage of post-deglutitive relaxation.

**Conclusion:**

The VFSS represents a fundamental examination in the management of the dysphagic patient; this investigation may be associated with manometry providing anatomical and functional informations.

## Introduction

Dysphagia consists in alteration of the swallowing mechanism, due to different pathological conditions, which can occur at different levels; dysphagia can be classified in oropharyngeal and esophageal, according to the level of the functional alteration [1]. The exact prevalence of dysphagia is unclear, even if some AA suggest that 15% of the elderly population is affected [2].

In the elderly patients, predisposing factors for dysphagia are related both to changes in swallowing physiology and to several diseases [3]. The physiology of swallowing changes with advancing age, due to reductions in muscle mass and connective tissue elasticity resulting in loss of strength [4] and range of motion [5]. Dysphagia may be also symptom of neurologic disease, head/neck and esophagus cancer and consequent therapies, connective tissue disorders, trauma, infection, or iatrogenic illness [6]. Swallowing disorders are a relevant health concern in elderly patients since they may lead to severe consequences, as increased risk of malnutrition, dehydration and pneumonia or airway obstruction [3,6]. Aging is still accompanied by a decline in the healthy function of all organs in response to several mechanisms like oxidative stress and elevated ROS (Reactive oxygen species) overproduction. Increased release of oxidant molecules due to several stress agents including drugs, diet and physical activity lead to cancer, diabetes, neurodegenerative, cardiovascular and other diseases [7-9]. The correct diagnosis of dysphagia and its aetiopathogenetic features requires a multidisciplinary approach. Between the various radiologic methods of investigation available, combined studies enabling simultaneous morphological and functional assessment, such as videofluoroscopy combined with manometry (VFMS-videofluoromanometric study), have taken on an increasingly front-line role in the diagnosis of dysphagia. Aim of this study is to analyze the main VFMSS findings in elderly dysphagic patients.

## Materials and methods

The VFMSS of 59 elderly dysphagic patients (32 women, 27 men, ranging in age from 68 to 89 years, mean 81 years) who undergone speech therapy assessment and videofluoromanometric (VFM) investigation of the swallowing process at our institution from January 2011 and December 2012, were retrospectively reviewed. The VFM study consisted of a parallel execution of videofluoroscopy (VFS) and manometry. The diagnostic team included a radiologist with experience in videofluoroscopy, a gastrointestinal surgeon with a good understanding of manometry and its application to the study of the pharynx and a speech therapist. A simultaneous manometric evaluation analyzed the tongue base pressure (the contact pressure between the posterior tongue thrust and the pharyngeal wall), upper esophageal sphincter (UES) tone (resting pressure, contraction pressure and residual pressure) and the bolus transit coordination. In contrast to the esophageal manometry that uses perfused catheters [10-12], in VFMSS a solid state catheter is used.

A Dyno Compact computerized system (MENFIS Biomedica s.r.l., Bologna, Italy) was used. This system was equipped with the following: (1) A graphics card for managing radiographic images; and (2) AVIUS-dedicated software, which enables digital-quality recording (PAL/NTSC, composite video or S-video) of the VFS study in AVI format with a 320 × 240 resolution and 25 Hz acquisition frequency. The concurrent pressure measurements were performed with a manometry catheter with endoluminal five-channel, solid-state microtransducers 2 cm apart at an angle of 120°-90°. The catheter was inserted through the nasal cavity into the stomach where, the value recorded was used for the calibration of 0. Afterwards, a pull through was performed and the catheter was withdrawn to allow for the positioning of the transducers. Transducer 1 was placed at Passavant's ridge to evaluate the correct closure of the rhinopharynx during swallowing and phonation. Transducers 2, 3 and 4 were placed in the pharynx. Transducer 5 was placed at the UES, and the correct placement was determined by the appearance of the characteristic M wave. During image acquisition, the video images and manometric trace were displayed in real time as a full screen image on the personal computer monitor. A cursor indicated the exact correspondence between the video images and the traces. Following the acquisition, the video and manometric trace could be analyzed during real-time reproduction or at reduced or increased speed, or it could be paused for a frame-by-frame analysis. The examinations were acquired with the patient standing or seated if the patient was unable to remain standing. VFM began with a baseline evaluation (without contrast) to study the motility of the vocal chords and soft palate. The VFM proceeded with barium contrast medium (Prontobario HD suspension, Bracco SpA, Milan; 250% w/v) at a dose of 5-15 mL that was optimized for the patient to evaluate swallowing. The patients were asked to hold the bolus in their mouth for several seconds and to swallow when asked by the operator. All phases of the process were video-recorded first in the anteroposterior and then the laterolateral view [1,13,14].

## Results and discussion

The availability of the combined videofluoroscopy and manometry swallowing study has increased our ability to investigate and interpret the pathological changes underlying the various types of dysphagia. Normal swallowing is a complex process in which several muscles and cranial nerves are involved [15] and swallowing alterations often are a result of several impairments, so they can appear as a complex syndrome with multiple findings rather than an isolated dysfunction [16], Moreover, apart from alteration of deglutition due to several pathologies, this physiologic function can be impaired by natural aging processes, leading to a typical pathophysiologic configuration known as "presbyphagia." [14,17]. Characteristic features of presbyphagia, such as weakness of pharyngeal ligaments, lower position of the hyoid bone, quadrangular shape of the valleculae, and expansion of the pharyngeal cavity, should not be considered abnormal findings but rather as paraphysiologic aspects of an aged deglutition system. The purposes of the dynamic radiologic study of the swallowing process are:

1. To define the normal anatomy of oropharyngeal region

2. To identify swallowing abnormalities (in the oral and pharyngeal phases)

3. To detect the mechanism responsible for the alteration

4. To determine the circumstances under which the patient can swallow safely

**Figure 1:**
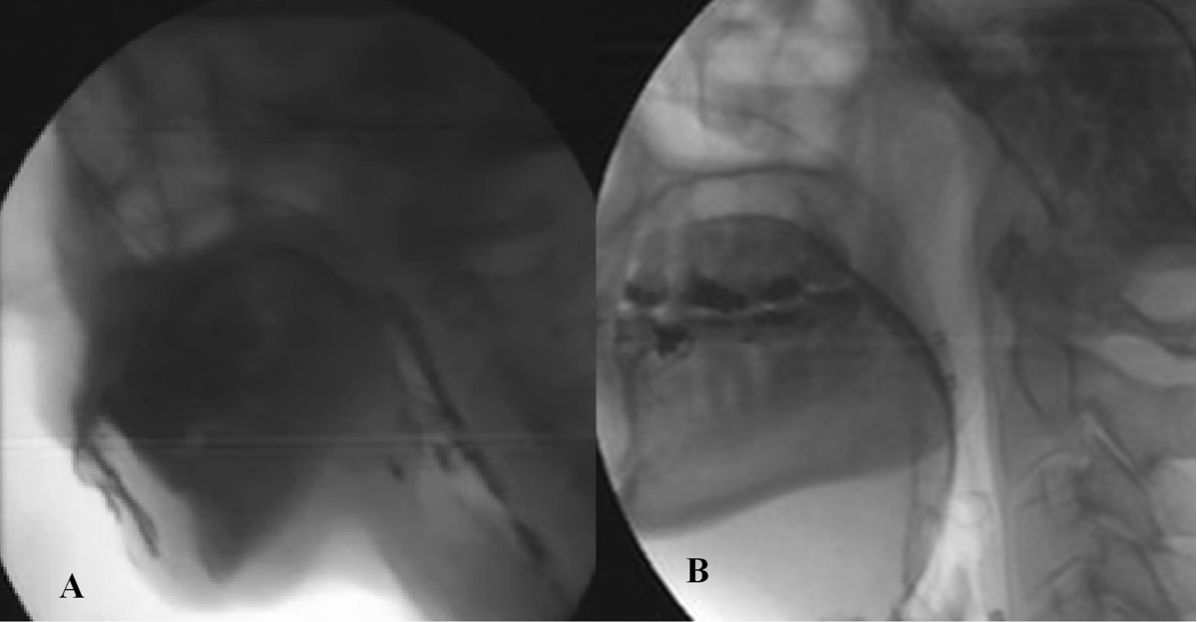
**Oral phase the preparation and the initial stage of swallowing should be explored by videofluoroscopy evaluating the ability to contain food in mouth (a) and to form a bolus and whether there is an inadequate convergence of Passavant's ridge with preswallowing aspiration (b)**.

**Figure 2:**
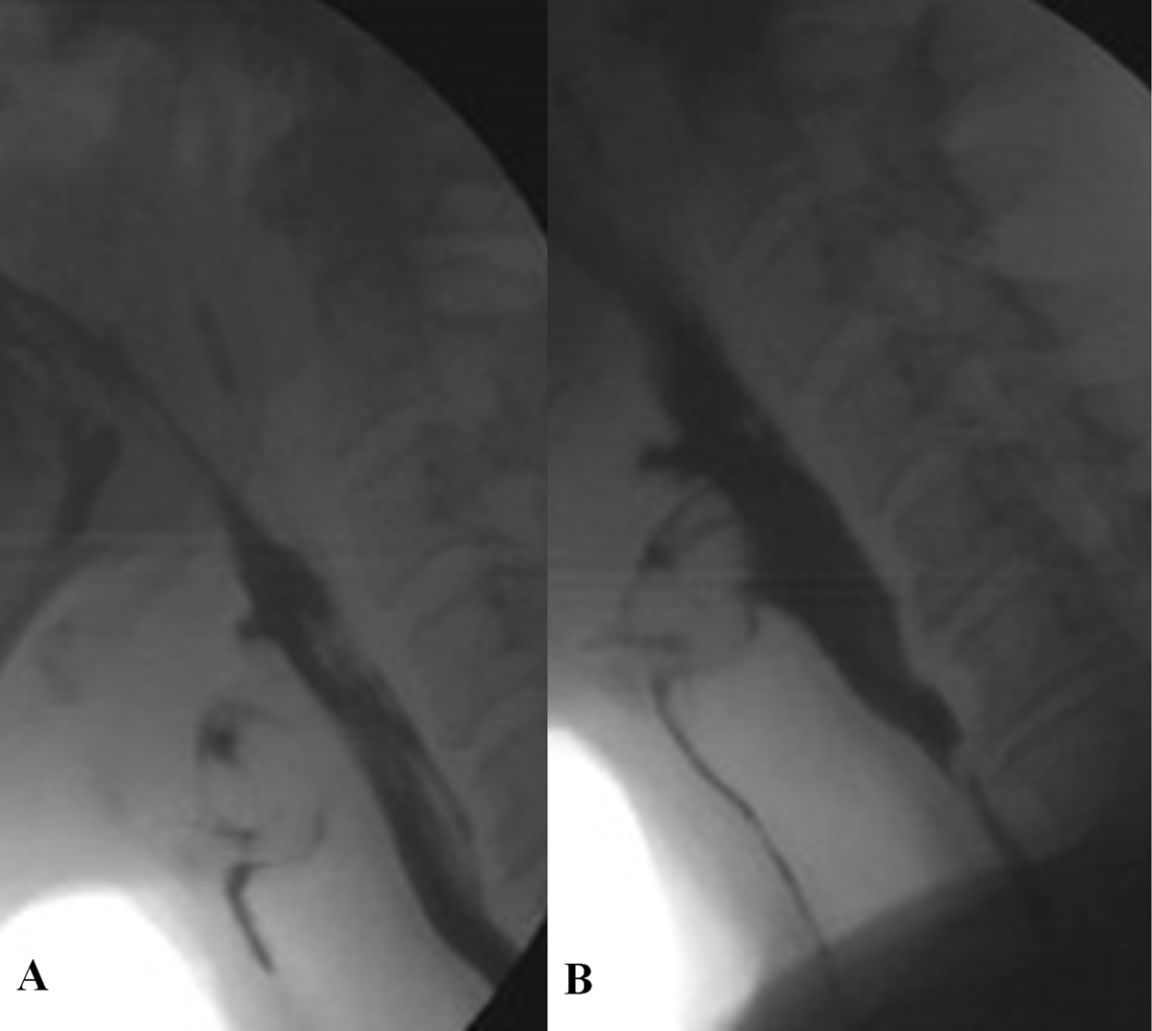
Pharyngeal phase at videofluoroscopy is possible evaluate if there is penetration (a) and/or aspiration (b) and the efficacy of laryngeal closure should be assessed too

**Figure 3:**
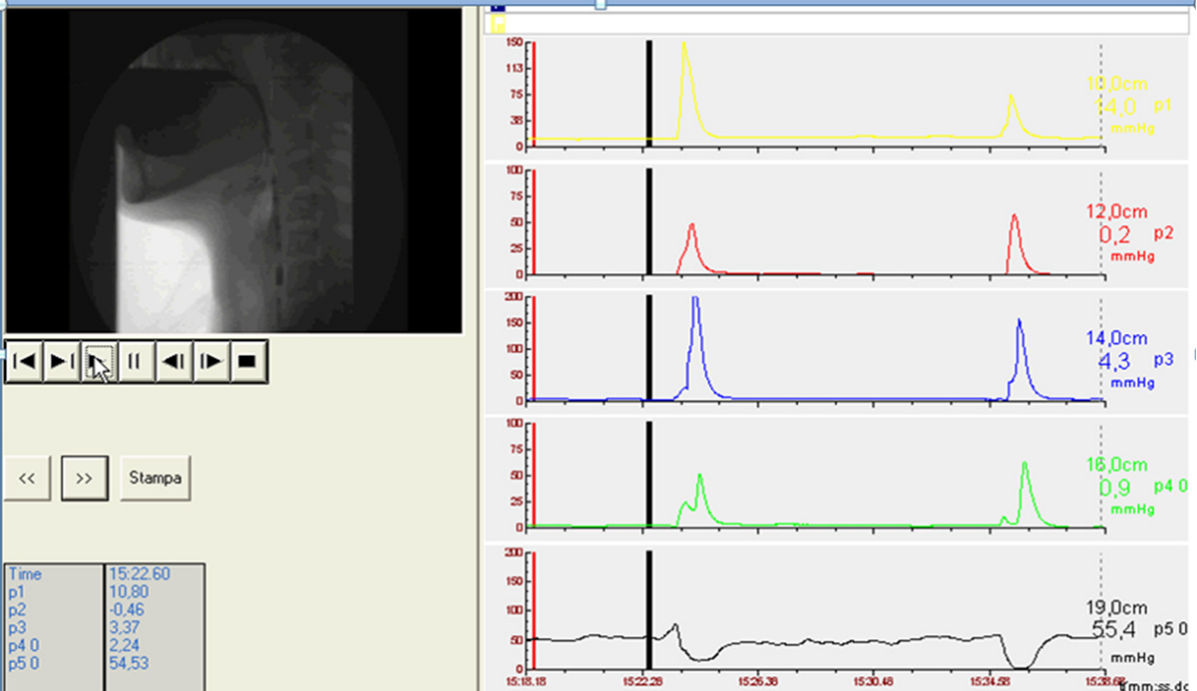
**The major manometric indicators at videofluomanometry are: proximal pharyngeal pressure (mmHg), distal pharyngeal pressure (mmHg), relaxation (Fig. 3) and coordination (Fig. 4) of upper esophageal sphincter (UES)**.

**Figure 4:**
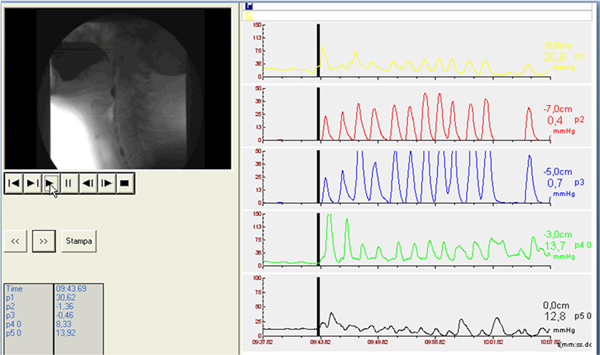
**The major manometric indicators at videofluomanometry are: proximal pharyngeal pressure (mmHg), distal pharyngeal pressure (mmHg), relaxation (Fig. 3) and coordination (Fig. 4) of upper esophageal sphincter (UES)**.

So, in the study of dysphagic patients the main findings to look for are the following: in the oral phase the preparation and the initial stage of swallowing should be explored by videofluoroscopy evaluating the ability to contain food in mouth and to form a bolus and whether there is an inadequate convergence of Passavant's ridge with preswallowing aspiration (Figure 1 a-b). In the pharyngeal phase is necessary to evaluate at videofluoroscopy if there is penetration and/or aspiration and the efficacy of laryngeal closure should be assessed too (Figure 2 a-b). The major manometric indicators are: proximal pharyngeal pressure (mmHg), distal pharyngeal pressure (mmHg), relaxation (Figure 3) and coordination (Figure 4) of upper esophageal sphincter (UES). In the esophageal phase is important to evaluate the oesophageal motility and the presence of peristalsis.

The manometric parameters used for LES were resting pressure, total length and percentage of post-deglutitive relaxation. The first 2 phases, lasting less than a second, need dynamic methods of study, whereas the esophageal phase can be studied with conventional methods [1]. Therefore, the precise definition of the anatomical-functional substrate responsible for the symptom influences the therapeutic approach. Clinically, patients affected by oropharyngeal dysphagia present symptoms characterized by difficulty in the initial phase of swallowing, such as cough, sense of choking associated with aspiration of food into the airways, nasal regurgitation and sialorrhoea. In contrast, when the anatomical-functional problem is located more distally - pharyngeal-oesophageal dysphagia - the patient experiences difficulty in the late swallowing phase with the sensation of food sticking in the lower part of the pharynx or chest, retrosternal pain, odynophagia and pyrosis [1]. Radiologic contrast examination is essential to evaluate physiologic swallowing dynamics and to detect pathologic impairments. Imaging features allow an accurate study of the tongue, palate, pharynx, and larynx, providing useful information for identifying the cause of the swallowing difficulty and for planning management. Only through the dynamic examination of swallowing is it possible to confirm or exclude the presence of food aspiration or penetration into the airways; this information influences the type of nutrition (oral or nonoral) the patient should receive [6].

## Conclusions

Currently, there is unanimity in considering the VFSS as a fundamental examination in the management of the dysphagic patient; this investigation may be associated with manometry providing anatomical, functional and clinical informations [18-29].

## Competing interests

The authors declare that they have no competing interests.

## Authors' contributions

AR: conceived the study, carried out the examinations, analyzed and interpreted the data, drafted the manuscript.

FI: conceived the study, carried out the examinations, analyzed and interpreted the data.

LDV: conceived the study, carried out the examinations, analyzed and interpreted the data.

LM: critically revised the manuscript.

DB: critically revised the manuscript.

GDG: critically revised the manuscript.

EAG: conceived the study and critically revised the manuscript.

MG: conceived the study, analyzed and interpreted the data and drafted the manuscript.

SC: conceived the study, analyzed and interpreted the data, critically revised the drafted manuscript.

All authors read and approved the final manuscript.

## Authors' information

AR: Post-Doctoral Fellow in Radiology at Second University of Naples

FI: Resident in Radiology Training Program at Second University of Naples

LDV: Resident in Radiology Training Program at Second University of Naples

LM: Resident in Radiology Training Program at Second University of Naples

DB: Resident in Radiology Training Program at Second University of Naples

GDG: Resident in Radiology Training Program at Second University of Naples

EAG: Associate Professor of Radiology at University of Cagliari

MG: Associate Professor of Radiology at University of Ferrara

SC: Associate Professor of Radiology at Second University of Naples

## Declarations

Publication of this article was funded by personal funds.

This article has been published as part of *BMC Surgery *Volume 13 Supplement 2, 2013: Proceedings from the 26th National Congress of the Italian Society of Geriatric Surgery. The full contents of the supplement are available online at http://www.biomedcentral.com/bmcsurg/supplements/13/S2
